# Modulation of Risky Choices in Recently Abstinent Dependent Cocaine Users: A Transcranial Direct-Current Stimulation Study

**DOI:** 10.3389/fnhum.2014.00661

**Published:** 2014-08-27

**Authors:** Alessandra Gorini, Claudio Lucchiari, William Russell-Edu, Gabriella Pravettoni

**Affiliations:** ^1^Department of Health Sciences, University of Milan, Milan, Italy; ^2^Applied Research Unit for Cognitive and Psychological Science, European Institute of Oncology, Milan, Italy; ^3^Library, European Institute of Oncology, Milan, Italy

**Keywords:** risk perception, drug addiction, cortical stimulation, reward, dorsolateral prefrontal cortex

## Abstract

Previous neurobiological and neuropsychological investigations have shown that risk-taking behaviors and addictions share many structural and functional aspects. In particular, both are characterized by an irresistible need to obtain immediate rewards and by specific alterations in brain circuits responsible for such behaviors. In this study, we used transcranial direct-current stimulation over the dorsolateral prefrontal cortex (DLPFC) of two samples of subjects (18 dependent cocaine users and 18 control subjects) to investigate the effects of left and right cortical excitability on two risk tasks: (1) the balloon analog risk task (BART) and (2) the game of dice task (GDT). All subjects randomly received a left anodal/right cathodal stimulation (LAn+), a right anodal/left cathodal stimulation (RAn+), and a sham (placebo) stimulation each run at least 48 h apart. Participants were asked to perform the BART and the GDT immediately before and after each stimulation. Our results reveal that the activation of the DLPFC (left and right) results in a reduction of risky behaviors at the BART task both in controls subjects and cocaine dependent users. The effect of tDCS on GDT, instead, is more complex. Cocaine users increased safe behavior after right DLPFC anodal stimulation, while risk-taking behavior increased after left DLPFC anodal stimulation. Control subjects’ performance was only affected by the anodal stimulation of the right DLPFC, resulting in an increase of safe bets. These results support the hypothesis that excessive risk propensity in dependent cocaine users might be due to a hypoactivation of the right DLPFC and an unbalance interhemispheric interaction. In conclusion, since risky decision-making seems to be, at least in part, responsible for maintenance and relapse of addiction, we argue that a neuromodulation-based approach could represent a valuable adjunct in the clinical treatment of addiction.

## Introduction

Drug addiction can be described as a persistent state characterized by loss of control over drug-seeking and the compulsive desire to use drugs for acute rewards regardless of whether they involve risk of aversive consequences (Hyman and Malenka, 2001). Neurobiological studies on addiction have shown an association between the rewarding and reinforcing effects of drugs and the activation of several areas in the mesocorticolimbic network, as well as an altered dopaminergic activity in frontal cortical areas including the dorsolateral prefrontal cortex (DLPFC), the orbitofrontal cortex (OFC), and the anterior cingulate cortex (ACC) (Di Chiara and Imperato, 1988; Carlezon and Wise, 1996; Wise, 1996; Breiter et al., 1997; Kauer and Malenka, 2007; Kalivas and O’Brien, 2008) caused by acute or chronic exposure to addictive drugs. In particular, it has been demonstrated that stimulant drugs, such as cocaine and amphetamines, induce a direct increase in dopamine levels within the mesocorticolimbic circuit that results in alterations in cortical neurotransmission and excitability responsible of the addictive behaviors (Wolf et al., 2004). However, most of the data have been obtained from animal studies. Conversely, the data on the role of dopamine on human addiction are extremely limited because of the lack of a reliable technique to study neurotransmission in the live human brain.

From a neuropsychological point of view, dysfunctions within these frontal and prefrontal cortical circuits cause impulsivity and abnormalities in decision-making, risk perception, cognitive evaluation of consequences and errors, and goal identification that are supposed to play a key role in compulsive drug-seeking behavior (Lubman et al., 2004; Fishbein et al., 2005; Garavan and Stout, 2005; Krain et al., 2006). In particular, several studies have shown significant behavioral impairments of risky decision-making tasks, such as the Iowa gambling task (IGT) and the balloon analog risk task (BART), in substance-dependent individuals [e.g., Wang et al. (2013); Balconi et al. (2014); Canavan et al. (2014); Hulka et al. (2014); Yan et al. (2014)].

Recent studies (Fecteau et al., 2007a,b; Boggio et al., 2009) have shown that non-invasive brain stimulation, such as transcranial direct-current stimulation (tDCS), is crucial in order to determine the neural mechanisms underlying decision-making processes, as it can produce a behavioral impact through the stimulation of a given cerebral area. tDCS-induced modulations of cortical excitability have been proposed as being able not only to affect human cognitive functions but also to modify addictive behaviors. In fact, an increase in cortical excitability of the DLPFC has been shown to be effective in temporarily reducing substance craving in a sample of chronic cigarette smokers (Fregni et al., 2008) and alcohol-dependent subjects (Boggio et al., 2008). These data are in accordance with previous studies showing that different kinds of non-invasive stimulations of the frontal cortex other than tDCS [i.e., transcranial magnetic stimulation (TMS) and repetitive TMS] reduce craving for some drugs such as nicotine (Eichhammer et al., 2003; Fregni et al., 2008) or cocaine (Camprodon et al., 2007).

From a neuropsychological point of view, it is known that tDCS applied to the frontal areas affects human reasoning as demonstrated by Kincses et al. (2004), who found that anodal stimulation of the left prefrontal cortex improves implicit probabilistic classification learning. Beeli et al. (2008) also showed that anodal stimulation of left and right DLPFC of car drivers leads to a more careful driving style in virtual scenarios indicating a modification in their risk-taking behavior. A conservative and risk-averse response style was also found in a sample of healthy volunteers during bilateral stimulation over the DLPFC using tDCS (Lejuez et al., 2002). In addition, Fecteau et al. (2007a) found that healthy volunteers receiving anodal stimulation over the right DLPFC coupled with cathodal stimulation over the left DLPFC preferred the safest prospects to the other available options as measured by the risk task. Conversely, participants receiving anodal stimulation over the left DLPFC coupled with cathodal stimulation over the right DLPFC did not differ in their choice related to risk-taking behaviors from those receiving sham stimulation.

Taken together, these results show that risk-taking behaviors and addictions share many structural and functional aspects (Bechara et al., 2000, 2002; Grant et al., 2000; Goeders, 2002; Lejuez et al., 2003; Epstein et al., 2007), and that non-invasive electrical stimulation of neural circuits implicated in risk taking and reward mechanisms can modify the need to obtain immediate reward, consequently reducing addictive behaviors (Fregni et al., 2007, 2008; Boggio et al., 2009). Within this framework, the main aim of this study was to compare the effect of the left and right tDCS over the DLPFC (An+ over the left DLPFC/Ca− over the right DLPFC and vice versa) on two different risk tasks in a sample of dependent cocaine users and control subjects. Consistently with previous research (Romero et al., 2010), we expected a reduction in risky behaviors consequent to the DLPFC stimulation, especially in cocaine users, who are known to have a DLPFC deficit.

In contrast to an earlier study on the modulation of risk behavior by external brain stimulation (Knoch et al., 2006), we used tDCS instead of TMS. Compared to TMS, tDCS has some significant advantages: it induces a stronger modulatory effect on brain activity (Nitsche and Paulus, 2001; Romero et al., 2002) while participants barely notice the stimulation; it allows the possibility to change the direction of the current flow so that neural excitability can be either enhanced or decreased; and, finally, it allows for a reliable sham condition (Gandiga et al., 2006).

## Materials and Methods

We conducted a single-blind, sham-controlled study to investigate the effect of tDCS over the left and right DLPFC on two decision-making tasks (risk tasks) in dependent cocaine users compared to control subjects.

The experiment was performed with the understanding and written consent of each subject. The study, approved by the ethical committee of the University of Milan, conformed to the ethical standards of the Declaration of Helsinki.

### Subjects

Thirty dependent cocaine users diagnosed according to the DSM-IV criteria for substance abuse, were recruited in a private hospital in the north of Italy, where they were hospitalized for a period of 4–6 weeks in order to withdraw from drugs. Only 18 patients out of 30 who met the following criteria were included in the trial: (1) primary diagnosis of substance abuse; (2) cocaine as only drug taken (as resulted from the drug concentration in the hair samples); (3) cocaine use of >0.5 g/month (over the past 6 months); (4) abstinence duration of at least 2 weeks before the test; (5) absence of other current or previous axis I DSM-IV psychiatric disorder; (6) no history of organic mental illnesses (migraine, headache, seizure disorder, head tumor, clinically significant head trauma, and vestibular abnormalities) or mental retardation; and (7) no family history of a severe DSM-IV psychiatric disorder such as schizophrenia, bipolar disorder, or obsessive–compulsive disorder. Both males (*N* = 10) and females (*N* = 8) aged between 18 and 50 years and proficient in Italian were included in the study. Since all patients had already been hospitalized for at least 2 weeks at the time of the evaluation, we could be sure that they were not using opioids, cannabis, and other illegal drugs or alcohol. In order to control and reduce irritability induced by drug abstinence, all patients underwent a sedative-based treatment (lorazepam) during the first week of admission to hospital. After that, benzodiazepines were gradually reduced, so that the sedation effect almost disappeared at the end of the second week (when they were selected to participate in the study). The computerized continuous performance test (CPT) (Conners, 2000) was administered before each experimental session in order to evaluate the patients’ attentive performance to be sure that they were able to participate to the trial.

Eighteen volunteers, non-abusers, matched with patients for gender and age, were recruited by advertisement to participate in the study as controls. The drug abuse screening test (DAST) (Gavin et al., 1989), one of the most widely used screening questionnaires for drug abuse and addiction, was administered to the control group in order to exclude the possibility that they were abusing or had abused any kind of illegal drugs (including cannabis) in the past. Control subjects were included in the study if they satisfied the two following criteria: (1) absence of a current or previous axis I DSM-IV psychiatric disorder; and (2) no history of organic mental illnesses (migraine, headache, seizure disorder, head tumor, clinically significant head trauma, and vestibular abnormalities) or mental retardation. Control subjects were also tested with the CPT before each experimental session.

Subjects did neither receive any financial compensation for participation nor did they receive any money collected during the tasks. At the moment of the experiment, all subjects were naive about tDCS and the risk tasks administered to them.

### Clinical assessment

Patients included in the study were assessed by independent clinicians (psychiatrists, MA-level chartered psychologists or Ph.D.-level chartered psychotherapists) who were not directly involved in the experimental trial.

#### Pre-test evaluation

The following psychometric questionnaires were administered to the subjects before the first tDCS session:

- Beck depression inventory (BDI-II) (Beck et al., 1996);
- Barratt impulsiveness scale (BIS-11) (Patton et al., 1995);
- DAST (Gavin et al., 1989) (only to the control group).

### Transcranial direct-current stimulation

Transcranial direct-current stimulation consists of applying constant direct current over the scalp by attaching electrodes of different polarities connected to a portable stimulator to the skin (Nitsche and Paulus, 2000, 2001; Iyer et al., 2005) (HDC-stim, Newronika, Milan, Italy). The electrodes are made of conductive rubber and put in saline-soaked synthetic sponges to prevent chemical reactions at the contact point between electrode and skin (Nitsche et al., 2003). The electrodes are thick (0.3 cm), rectangular, with a size of 32 cm^2^, which results in a current density of 0.03–0.08 mA/cm^2^ when used with a current of 1.5 mA.

In the present study, we used a controlateral stimulation, which means that all participants received a left anodal/right cathodal stimulation (LAn+), plus a right anodal/left cathodal stimulation (RAn+), and plus a sham (placebo) stimulation. The three stimulations were administered at least 48 h apart. The three types of stimulation were performed in counterbalanced order across subjects. For the LAn+ stimulation, the anode electrode was placed over the DLPFC in correspondence of the left F3 (according to the 10–20 EEG international system), while the cathode electrode was placed over the right F4. For the RAn+ stimulation, the polarity was reversed: the anode electrode was placed over the right F4 and the cathode electrode was placed over the left F3. We opted for a contralateral stimulation since it has been demonstrated to be more effective than the unilateral stimulation in modulating risk-taking behavior in risk tasks (Fecteau et al., 2007a). Consistently with previous studies (Fumagalli et al., 2010), each stimulation session lasted 20 min and was immediately preceded and followed by the risk tasks. The same procedure was used for the sham stimulation, except for the fact that current was applied only for the first 30 s, according to a method that has been shown to be reliable for blinding subjects with respect to the stimulation condition (Gandiga et al., 2006).

### Experimental procedure

All subjects (patients and controls) underwent the three stimulation blocks (LAn+, RAn+, and sham) in a randomized order (across subjects and groups).

Participants sat on a comfortable chair in front of a computer screen and were asked to perform the two risk tasks before each stimulation (baseline) (the order of the task was also randomized). Once completed, they received a 20-min stimulation (or sham), and were then asked to perform the same two tasks again (post-stimulation). In addition, during the first experimental session all subjects were asked to complete the psychometric questionnaires.

#### Risk tasks

##### Balloon analog risk task

**Figure 1:**
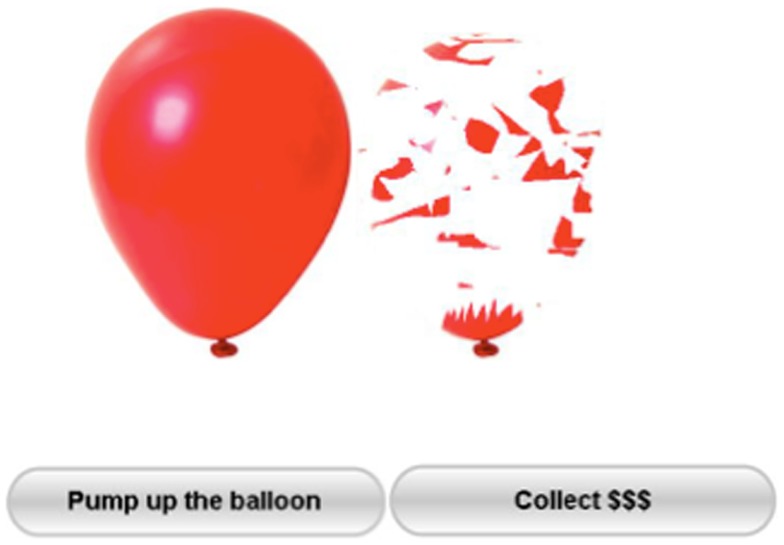
**The BART test**. Participants press the button “pump up the balloon” to inflate puffs of air into a balloon presented on a computer screen. Every successful pump adds 5 cents to their temporary bank for that trial. If the balloon explodes before the participant presses the “collect $$$” button then nothing is won on that trial.

The BART (Figure 1) is a computerized test that involves actual risky behavior for which, as is often the case in real-world situations, riskiness is rewarded up until a point at which further riskiness results in poorer outcomes. Unlike in other behavioral measures of risk taking that have consistently shown a poor convergent validity with self-report measures of risk-related constructs (Bentler and McCain, 1976; White et al., 1994; Stuart, 1998; Mitchell, 1999; Petry, 2001) and a limited relationship with the range of risk behaviors occurring outside the laboratory (Jessor and Jessor, 1977; Gullone and Moore, 2000; Pack et al., 2001), BART is significantly correlated with scores on self-report measures of risk-related constructs and with the self-reported occurrence of real-world risk behaviors (Lejuez et al., 2002). Subjects’ performance at the BART test seems to be significantly related to the activity of the DLPCF as shown by Sela et al. (2012).

Balloon analog risk task consists of presenting subjects with a small-simulated balloon on a computer screen. As shown in Figure 1, the balloon is accompanied by a balloon pump, a reset button labeled “collect $$$,” a permanent money-earned display labeled “total earned,” and a second display listing the money earned on the last balloon and labeled “last balloon.” Every time the subject clicks on the pump he/she inflates the balloon temporarily collecting 5 cents.

When a balloon is pumped past its individual explosion point, it explodes and all of the money in the temporary bank is lost, while the next uninflated balloon appears on the screen. At any point during each trial, the participant can stop pumping the balloon and click the “collect $$$” button in order to transfer all money to his/her permanent bank. Should the balloon pop, the participants would lose all money accrued for that trial. Participants were given no specific information regarding the likelihood of the balloon exploding. Each balloon was set to explode on a variable ratio, with the mean number of pumps before explosion set at 64; consequently, the probability that the balloon would explode on the first pump was 1/128, on the second pump was 1/127, and so on, with an increasing risk of balloon explosion along time (Lejuez et al., 2002). An individual balloon trial could be discontinued at any point, and the money accrued for that respective trial would be collected in a reserve. A total of 20 balloons were presented to each subject. The primary score used to measure BART performance is the adjusted average number of pumps on unexploded balloons, with higher scores indicative of greater risk-taking propensity. This score is preferred as dependent measure as it is not constrained by the pseudo-random popping threshold of the balloon.

##### Game of dice task

**Figure 2:**
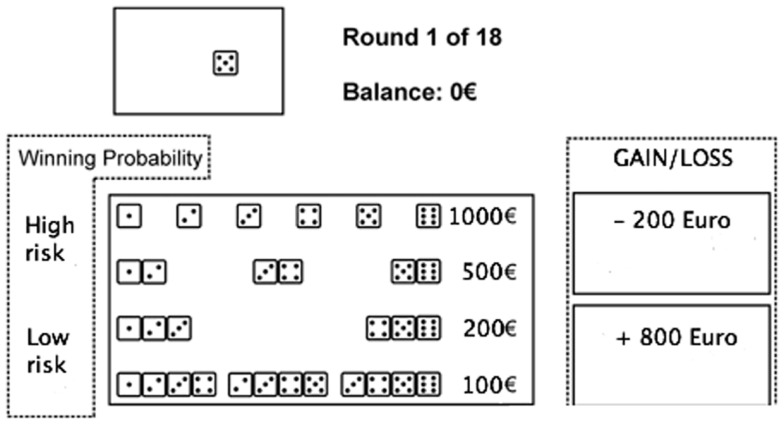
**The game of dice task**. Before each trial, subjects have to decide between a single number, a combination of two numbers, three numbers, or four numbers. Thereafter, the dice are tossed (animated on the screen) and the result is presented. At the same time, an acoustic signal indicates whether the throw was successful or not, and the gain or loss is shown. Furthermore, the amount of capital changes depending on the received gain or loss, which is shown immediately.

In the game of dice task (GDT) (Figure 2) rules for reinforcement and punishment are explicitly expressed to the subjects while the outcome is defined by uncovered probabilities (Brand et al., 2005a). As shown by Brand et al. (2005b) performance in the GDT is significantly related with DLPFC functioning.

In the GDT, subjects are asked to maximize their fictive starting capital (1000 €) within 18 dice throws. One virtual single dice and a shaker are used. In each trial, subjects have to bet on the number that will occur in the next throw. They can choose between the six different single numbers or a combination of two, three, or four numbers. Each choice is associated with specific fictive gains and losses depending on the probability of occurrence of choice: 1000 € gain/loss for the choice of a single number (winning probability 1:6), 500 € gain/loss for two numbers (winning probability of 2:6), 200 € gain/loss for three numbers (winning probability 3:6), and 100 € gain/loss for four numbers (winning probability 4:6) (see Figure 2). Visual and acoustic stimuli give feedback to the participants, while the total amount of earned capital changes according to the subject’s winning and losses.

### Statistical analysis

Pearson coefficient was use to explore correlations between-subjects’ variables. *T*-tests were used to compare groups on baseline parameters. Performances at the two risk tasks before and after each stimulation were calculated using a mixed-design ANOVA with stimulation type (sham × LAn+ × Ran+) and stimulation condition (pre × post) as within-subjects factors and group (cocaine users × controls) as between-subjects factor. Simple effects analyses were used to compare the variables levels. For all the ANOVAs, Bonferroni correction for multiple comparisons was applied. All statistical analyses were performed using SPSS 21.

## Results

### Demographic characteristics of the two groups

The patient and control groups did not differ either in gender composition (both patients and controls were 10 males and 8 females) or in age (patients: mean age = 38.4 years, SD = 7.5; control subjects: mean age = 36.8 years, SD = 7.8) or educational level (patients: mean education = 14.42 years, SD = 2.3; control subjects: mean education = 15.56 years, SD = 1.9) (see Table 1).

**Table 1:** **Means and mean total scores (±SD) of descriptive group characteristics and ratio of participants gender**.

	Cocaine users	Controls	*p*
*N*	18	18	
Age (M, SD)	38.4 (8.2)	36.8 (7.8)	0.366
Age range	29–53	24–50	
Gender (M:F)	10:08	10:08	
Education (years)	14.42 (2.3)	15.56 (1.9)	0.410
Years of drug use	12.63 (6.4)		
Hair cocaine level (ng/10 mg hair)	432.92 (42.4)		
No. of weekly doses (1 dose = 100 mg)	58.8 (4.3)		
No. of smokers	16:2	13:5	
No. of cigarettes a day (mean)	8.6	7.8	0.345

### Self-reported and objective drug use

Self-reported drug use showed that dependent cocaine users used cocaine on a regular basis with a mean weekly consumption of more than 5 g of cocaine. Results from the hair toxicology analyses performed when patients were admitted to the hospital revealed that self-reported cocaine use (gram per week, cumulative dose, and duration of use) corresponded with concentrations of cocaine and its metabolites in the hair samples (*r* = 0.28, *p* < 0.01). Such analysis confirmed that for cocaine users enrolled in the study, cocaine had been the only drug of use over the past 6 months.

Cocaine was used for a mean of 12.63 years (SD = 6.4), while the mean time of drug abstinence after admission to the hospital was 16 days (SD = 2).

### Continuous performance test

Results from CPT showed no significant differences between patients and control subjects as shown in Table 2.

**Table 2:** **Conners’ continuous performance test-second edition (CPT-II)**.

	Commission index	Omission index	Response style index	Hit reaction time	Hit reaction time standard error	Detectability index
Controls	48.75 (SD 12.14)	44.24 (SD 3.52)	47.67 (SD 8.50)	45.67 (SD 10.02)	39.45 (SD 8.12)	51.12 (SD 10.54)
Dependent cocaine abusers	49.45 (SD 15.43)	48.33 (SD 5.64)	49.12 (SD 6.34)	46.12 (SD 11.00)	41.54 (SD 6.13)	53.13 (SD 12.20)
Significant difference	No	No	No	No	No	No

### Impulsivity and depression

We analyzed whether impulsivity score measured by the BIS-11 scale had any effect on the subjects’ performance in the risk tasks before any stimulation occurred (baseline). A *t*-test for independent groups showed that, as expected, patients were characterized by a higher level of impulsivity compared to control subjects [patients’ score = 61.5; controls’ score = 55.3; *t*(34) = 2.156, *p* < 0.005]. However, this difference did not significantly correlate with the subjects’ performance (risk behaviors and response time) in the two risk tasks at the baseline condition. If this were the case, we could attribute any differences found in risk-taking behaviors to this specific personality trait instead of to the different brain stimulations (Crews and Boettiger, 2009).

Regarding the BDI-II no significant differences were found between the two samples (patients’ score = 5.9 ± 2.6; controls’ score = 6.8 ± 2.3), indicating that abusers were not significantly more depressed than controls (see Table 3).

**Table 3:** **Group mean total scores (±SD) and comparisons (*t*-test) of depression, impulsivity, and baseline performances (absolute first trial) at risk tasks**.

	Cocaine users	Controls	*p*
**DEPRESSION**			
BDI-II	5.9 (2.6)	6.5 (2.3)	0.886
**IMPULSIVITY**			
BIS-11 (total)	61.50 (6.2)	55.31 (4.5)	0.042
BIS-11 (attentional)	16.19 (3.2)	14.96 (2.2)	0.089
BIS-11 (motor)	17.56 (2.4)	16.25 (4.1)	0.445
BIS-11 (non-planning)	27.75 (2.1)	24.10(2.9)	0.012
**BASELINE RISK PERFORMANCES**			
Baseline at BART task	23.12 (4.7)	20.91 (3.6)	0.213
Baseline at GDT task	12.85 (2.1)	15.05 (2.3)	0.012

*Average adjusted pumps are reported for BART and average safe bets (choices in line 3 or 4) for GDT*.

### Task performance

At the baseline condition (i.e., the absolute first trial in each task), the two groups’ performances were not statistically different at the BART [*t*(34) = 1.3123, *p* = 0.213], while a significant difference was found at the GDT [*t*(34) = −3.314, *p* < 0.005], regarding the number of safe bets (see Table 3).

In order to analyze the effects of the tDCS on risk modulation, we compared the performances within and between-subjects on the GDT and the BART test before and after each stimulation (LAn+, RAn+, and sham) using a mixed-design ANOVA with group (patients vs. controls) as between-subject factor, and condition (pre vs. post) and stimulation (LAn+, RAn+, sham) as within-subject factors.

**Figure 3:**
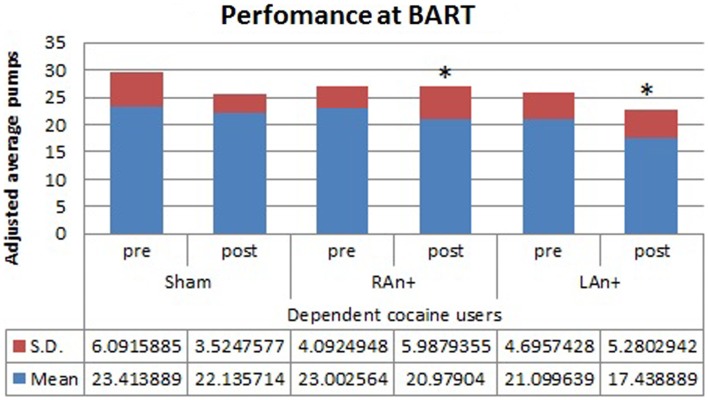
**Cocaine users’ performance at the BART test**. This figure represents the cocaine users’ performance (adjusted average pumps) at the BART test before and after each stimulation (*p < 0.05).

**Figure 4:**
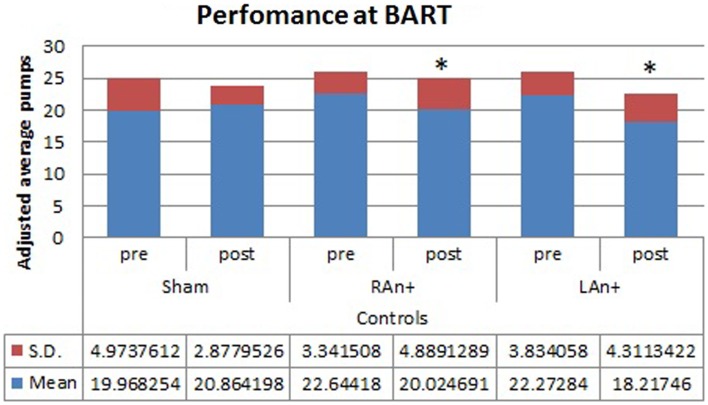
**Controls’ performance at the BART test**. This figure represents the controls’ performance (adjusted average pumps) at the BART test before and after each stimulation (*p < 0.05).

Regarding the BART test, we found a significant interaction between condition and stimulation [*F*(2,68) = 4.633, *p* < 0.05], while no significant main effects were found [*F*(1,34) = 0.354, *p* = 0.543 for condition; *F*(2,68) = 1.164, *p* = 0.353 for stimulation; *F*(1,34) = 0.989, *p* = 0.567 for group]. Simple effects analysis was used to investigate these differences showing that the sham stimulation did not produced any statistical difference [*F*(1,34) = 0.81, *p* = 0.876] in subjects’ behavior. At the opposite, LAn+ and RAn+ induced a significant decrease of risk-taking behaviors [LAn+: *F*(1,34) = 11.531, *p* = 0.002; RAn+: *F*(1,34) = 9.931, *p* = 0.038] (see Figures 3 and 4).

Considering performances at the GDT test, we found a significant interaction between condition and stimulation [*F*(2,34) = 3.456, *p* < 0.05], and between group × condition × stimulation [*F*(2,34) = 4.345, *p* < 0.05], while no significant main effects were found [*F*(1,34) = 0.831, *p* = 0.241 for condition; *F*(2,68) = 0.994, *p* = 0.553 for stimulation; *F*(1,34) = 1.389, *p* = 0.478 for group].

**Figure 5:**
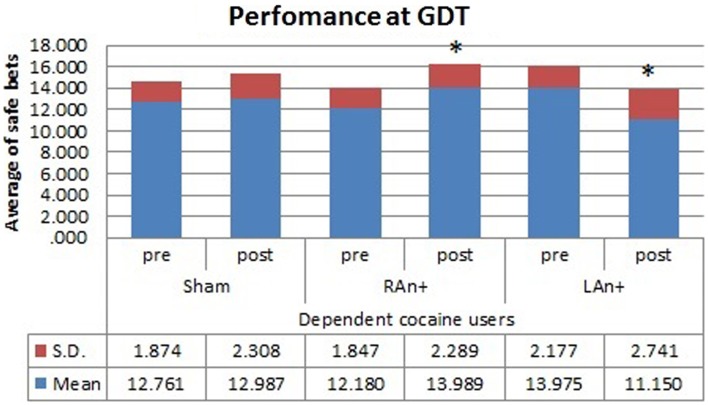
**Cocaine users’ performance at the GDT test**. This figure represents the cocaine users’ performance (average of safe bets) at the GDT test before and after each stimulation. Safe bets are defined as choices in line 3 and 4 (*p < 0.05).

**Figure 6:**
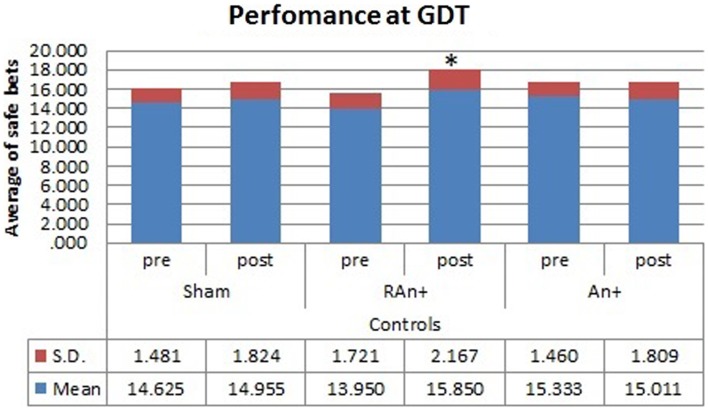
**Controls’ performance at the GDT test**. This figure represents the controls’ performance (average of safe bets) at the GDT test before and after each stimulation. Safe bets are defined as choices in line 3 and 4 (*p < 0.05).

Simple effects analysis revealed that a statistical difference between pre- and post-stimulation was present, in the patient group, both in the RAn+ [*F*(1,34) = 8.037, *p* = 0.008] leading to an increase of conservative bets (safe behavior), and in the LAn+ [*F*(1,34) = 6.691, *p* = 0.037] where risky choices increased (risk-taking behavior), but not in the sham condition [*F*(1,34) = 1.117, *p* = 0.335]. Regarding control subjects a significant increase of safe bets was found only in the RAn+ [*F*(1,34) = 6.564, *p* = 0.020], while LAn+ [*F*(1,34) = 1.985, *p* = 0.435] and sham [*F*(1,34) = 0.987, *p* = 0.752] did not induce any significant change (see Figures 5 and 6).

## Discussion

Risk-taking behaviors involve the potential for danger or harm while providing an opportunity to obtain different forms of reward (Leigh, 1999). Some of these behaviors, such as drug dependence, place individuals at risk for deleterious health outcomes. For this reason, many researchers have attempted to better understand this phenomenon in order to prevent, and where possible treat, the negative outcomes associated with risk taking.

Using tDCS to stimulate the DLPFC, which is known to be implicated in addictive behaviors and reward mechanisms, we analyzed the potential of cortical non-invasive stimulation to modulate risk-taking behaviors in dependent cocaine users and control subjects.

First of all, we found that, in our sample, impulsivity, which is often considered a relevant source of abusers’ risk-taking behaviors, has no significant effect on subjects’ choices. In fact, unlike other studies [e.g., Lejuez et al. (2002)], which found a significant relationship between the BART score and the impulsivity level, and despite the fact that, also in our sample, cocaine users are characterized by a higher level of impulsivity compared to healthy subjects, we only found a slight, non-significant negative correlation between impulsivity and the subjects’ performance at the BART, and a general lack of correlation between impulsivity and response time. We may thus infer that risky behaviors (such as substance use and abuse) are also mediated by different factors (i.e., deficits in decision-making abilities) other than impulsivity.

Analyzing the effects of the cortical stimulation on the BART task, we found a significant decrease of risk taking after stimulations of both the left and the right DLPFC, both in cocaine users and controls. Such data confirm the results obtained by Fecteau et al. (2007a) and support the notion that the interhemispheric balance of activity across the DLPFCs is critical in decision-making tasks involving the decision to stop taking risks in the presence of secure chances to obtain a reward. Furthermore, considering the effect of stimulation over the rDLPFC in controls, our results are consistent with previous results in which this area was found to modulate risk decisions. In particular, van’t Wout et al. (2005) reported a specific alteration of strategic decisions in the ultimatum game (UG) using repetitive TMS over the rDLPFC. From a cognitive point of view, the BART task is easier than the UG and does not involve strategic evaluations, and this suggests that the rDLPFC is probably implicated in a general risk evaluation process. When undertaking a more complex task, such as the GDT, in which subjects have to take into consideration not only the immediate risk of losing money but also the probability associated with each possible outcome, or combination of outcomes, we observed a different pattern of responses in abusers and controls. In fact, while the activation of the right DLPCF (RAn+) led both abusers and controls to increase their safe choices, left stimulation (LAn+) only affected patients’ performance, leading to a more risk-taking behavior.

These results support the hypothesis that excessive risk propensity in cocaine users, especially in situations that require articulated decision-making processes with explicit risk-related rules, is probably due to a hypoactivation of right DLPFC as demonstrated by the fact that the excitatory anodal stimulation of this area causes a significant decrease of risky bets in all subjects. These data confirm a general role of rDLPFC in risks evaluation and regulating risk-taking/risk-avoiding behavior. Since we also found a significant effect of the anodal stimulation over the left DLPFC (LAn+) only in cocaine users, we may suggest that also the interplay between right and left DLPFC might be impaired in these subjects. However, this data require further investigations, for instance, by the use of neuroimaging techniques.

The present study has some limitations. The major methodological limitation regards the tDCS spatial resolution. Given the electrode size of 32 cm^2^ the spatial resolution is necessarily low since the spread of current from the stimulated region to neighboring and interconnected areas is very likely (Ilmoniemi et al., 1997; Bestmann et al., 2005). For this reason, we expect that the stimulation of the DLPFC induces a simultaneous effect in other prefrontal regions such as the ventromedial and OFC, which may consequently influence the subjects’ performance. Thus, despite this well-known methodological problem, there are several studies supporting the usability of tDCS (Nitsche et al., 2003; Uy and Ridding, 2003) for the stimulation of specific brain areas. Another important limitation that can affect the external validity of our data is the small number of patients included in the study. This was mainly due to the extreme difficulty of recruiting abstinent patients with a primary diagnosis of substance abuse and with no other neurological or psychiatric disorders. Nevertheless, compared to the previous tDCS studies, the present protocol has the advantage of being based on a mixed design in which each subject (cocaine users and control subjects) underwent the three experimental conditions (anodal–cathodal, cathodal–anodal, and sham stimulation). This approach is particularly useful when small samples are used in order to analyze both the within- and the between-subjects differences. Finally, we could neither assess the effects of dTCS stimulation on craving nor could we analyze the interaction between craving and other variables in modulating risk-taking before and after dTCS.

In conclusion, the present study supports the hypothesis that dependent cocaine users have functional abnormalities in the prefrontal neural networks involved in decision-making and risk-taking behaviors. In particular, our data suggest a single session of brain stimulation could be used to transiently modulate risk-taking behavior, and possibly even the drug-seeking process. However, since we have found a mismatch between LAn+ and RAn+ effect on cocaine users behavior, caution should be used in testing such interventions.

To date, available treatment options for addictive behaviors are limited, and long-term success rates are poor (O’Brien, 2008). Because risky decision-making seems to be, at least in part, responsible for the maintenance and relapse of addiction, a neuromodulation-based approach to modulate decision making, and executive functions in general, is particularly interesting and could ultimately represent a valuable adjunct in the clinical treatment of addiction. However, the long-term efficacy of such interventions should be assessed in future longitudinal studies.

## Conflict of Interest Statement

The authors declare that the research was conducted in the absence of any commercial or financial relationships that could be construed as a potential conflict of interest.
